# Anti-spike Antibody Status in Pre-vaccinated Healthy Participants and Rheumatoid Arthritis Patients During the Third Wave of COVID-19

**DOI:** 10.7759/cureus.37316

**Published:** 2023-04-09

**Authors:** Madhavi Eerike, Raja Sundaramurthy, Ravi Gandham, Varatharajan Sakthivadivel, Aravind Amshala, Rekha Priyadarshini, Anand K Pyati, Parag Patil

**Affiliations:** 1 Pharmacology, All India Institute of Medical Sciences, Bibinagar, Telangana, IND; 2 Microbiology, All India Institute of Medical Sciences, Bibinagar, Telangana, IND; 3 Internal Medicine, All India Institute of Medical Sciences, Bibinagar, Telangana, IND; 4 Pharmacy, All India Institute of Medical Sciences, Bibinagar, Telangana, IND; 5 Biochemistry, All India Institute of Medical Sciences, Bibinagar, Telangana, IND; 6 Laboratory Medicine, All India Institute of Medical Sciences, Bibinagar, Telangana, IND

**Keywords:** healthy participants, neutralizing antibodies, rheumatoid arthritis, pre-vaccination, anti-spike antibodies

## Abstract

Introduction

Anti-spike severe acute respiratory syndrome coronavirus 2 (SARS-CoV-2) antibodies produced after infection with the coronavirus disease of 2019 (COVID-19) will offer protection and prevent re-infection for a few months. Seroprevalence studies measuring the SARS‑CoV-2 immunoglobulin G (IgG) levels will be helpful to know the herd immunity level that prevents community transmission. Very few studies have addressed the antibody titer among healthy participants and rheumatoid arthritis (RA) patients. The present study was conducted to determine the anti-spike SARS-CoV-2 antibody (Ab) status before COVID-19 vaccination in healthy participants and RA patients.

Methodology

A cross-sectional study was conducted at a tertiary care hospital to estimate the serum anti-spike antibody levels against COVID-19 among the pre-vaccinated healthy participants and patients with RA during the third wave of COVID-19. After receiving written informed consent, participants were recruited as per the inclusion and exclusion criteria. Demographic details, co-morbid status, and medication details were collected. Five milliliters of blood samples were collected, and anti-spike antibodies were estimated. The SARS-CoV-2 Ab positivity rate was expressed in percentage and was correlated with gender and age groups. Ab-positive participants were classified into three categories based on the neutralizing antibody titers (NAT).

Results

A total of 58 participants (49 healthy volunteers and nine RA patients) were recruited. Out of 58 participants, 40 were males, nine were females among healthy participants, and one male and eight females in the RA group were enrolled. Among the RA patients, one participant was found to have the chronic obstructive pulmonary disease (COPD), and two participants with hypothyroidism. Antibody positivity was found to be 83.6% among the healthy volunteers and 100% in the RA patients. About 48% had NAT between 50 and 90%. There was no significant difference for age and gender-specific positivity for SARS-CoV-2 neutralizing antibodies and neutralizing antibody titers among healthy participants.

Conclusion

Our study showed 84% positivity for anti-spike SARS-CoV-2 antibodies around the third wave (between November 2021 and February 2022). The majority had high neutralizing antibody titers. The probable reason for the SARS-CoV-2 antibody positivity before vaccination was either asymptomatic infection or herd immunity.

## Introduction

Globally, 60 crores population was infected, and 65 lakhs syndrome coronavirus 2 (SARS-CoV-2) naturally produces antibodies (Ab) to several structural proteins of the virus, such as spike (S) and nucleocapsid proteins (N) [1]. The spike protein consists of two subunits, S1 and S2. The S1 subunit contains the receptor binding domain (RBD). Anti-spike antibodies are more effective than antibodies against nucleocapsid proteins in providing protection against SARS-CoV-2 infection.

The anti-spike severe acute respiratory syndrome coronavirus 2 (SARS-CoV-2) antibodies (Ab) produced after infection will offer protection and prevent reinfection for a few months, but the duration of protection is unknown [2]. Vaccination is an important measure to protect people against the pandemic. In India, the vaccination drive began on January 16, 2021, and more than a hundred crore COVID-19 vaccine doses administration was completed by October 21, 2021 [3]. At present, commercial SARS-CoV-2 serological assays shall detect the antibodies that are specific to these viral proteins/domains, but these tests cannot differentiate the induced anti-spike antibodies between past infections and vaccines.

Seroprevalence studies measuring the SARS‑CoV-2 immunoglobulin G (IgG) levels will be helpful to know the herd immunity level that prevents community transmission. Most of the seroprevalence studies have been carried out among infected individuals or health care workers or among the vaccinated population.

Herd immunity with neutralizing antibodies (NAbs) will prevent community transmission. Very few studies have addressed the antibody titer among healthy participants and rheumatoid arthritis (RA) patients. RA patients have an inherently heightened susceptibility to developing infections, including COVID-19, and this could be due to their treatment with immunosuppressants, such as corticosteroids and methotrexate.

The present study was conducted to determine the pre-vaccination anti-spike antibody status and protection level against COVID-19 before vaccination in healthy participants and RA patients who had never taken any dose of the COVID-19 vaccine. This study was part of an intramural study.

To estimate the anti-spike antibodies in pre-vaccinated healthy participants and in RA patients who had never taken any dose of the COVID-19 vaccine. To correlate the SARS-CoV-2 Ab positivity rate and neutralizing antibody titer (NAT) with age and gender.

## Materials and methods

A cross-sectional study was conducted at a tertiary care hospital to estimate the serum anti-spike antibody levels against COVID-19 among the pre-vaccinated healthy participants and patients with RA during the third wave of COVID-19, i.e., between November 2021 and February 2022. The study was conducted after receiving approval from the ethics committee (AIIMS/BBN/IECIAUG/2021/60). This is part of an intramural project where the effect of vaccination on clinical and immunological responses in autoimmune patients would be studied. In this study, the Ab status before vaccination was assessed.

Study population

Controls were healthy participants and the test group was composed of patients with rheumatoid arthritis on treatment with disease-modifying anti-rheumatic drugs (DMARDs). The participants included in the study had never taken the COVID-19 vaccine before the recruitment and without a history of symptomatic COVID-19 infection within the last three months at the time of recruitment. They were tested for COVID-19 by rapid antigen testing (RAT) before enrolling in the study.

Sample size

The sample size for the project was calculated based on the proportion of people with an adequate antibody response after COVID-19 vaccination in the RA group, which was 90.2% and the non-RA group, which was 97%. With a 95% confidence interval and 80% power, the calculated sample size was 96 (calculated 48 test group and 48 control group).

Sampling method

The control population was recruited from the vaccination center of our hospital after screening for their eligibility. RA participants were recruited from the general medicine department. After obtaining written informed consent, the participants were recruited as per the inclusion and exclusion criteria (Table 1).

**Table 1:** Inclusion and exclusion criteria. RA: rheumatoid arthritis, DMARDs: disease-modifying anti-rheumatic drugs.

Inclusion criteria	Exclusion criteria
Age >18 years both sex	Cancer, other autoimmune/immuno-compromised patients
Willing to give consent	Patients with active COVID-19 infection
Intend to receive the COVID-19 vaccine	Pregnant or breastfeeding women
Rapid antigen test (RAT)-negative	Hospitalized/critically ill patients
For RA patients-above criteria plus	
Diagnosed case of RA	
Both seropositive and seronegative RA	
On treatment with DMARDs (single or combination)	

From all the participants, demographic details, co-morbid status, and medication details were collected. Later, 10 ml of blood samples were collected by the experienced phlebotomist. The complete blood count (CBC), erythrocyte sedimentation rate (ESR), C-reactive protein (CRP), liver function test (LFT), and renal function test (RFT) were performed for all participants, and additionally, RA factor positivity and disease activity score-28 (DAS-28) were recorded for RA patients. The separated serum was used for estimating the anti-spike antibodies as per the instructions given in the kit (J. Mitra & Co. Pvt Ltd, India).

Anti-spike ab testing

COVID-19 Neutralizing Antibody Microlisa (J. Mitra & Co. Pvt Ltd, India) test is an enzyme immunoassay designed for semi-quantitative detection of neutralizing antibodies developed against SARS-CoV-2 in human serum/plasma that prevents the interaction between receptor binding domain (RBD) viral spike glycoprotein and cell receptor angiotensin-converting enzyme-2 (ACE-2) based on a “blocking assay.”

Specimen and controls are pre-incubated with horseradish peroxidase (HRP)-conjugated recombinant SARS-CoV-2 RBD protein in a tube and added to the microtitre wells coated with recombinant hACE2 protein, incubated and then washed to remove unbound HRP-RBD-neutralizing antibodies complex. Finally, a substrate solution containing chromogenic and hydrogen peroxide is added to the wells and incubated. A blue-colored reaction is stopped by a stop solution. An enzyme-substrate reaction is read by an enzyme immunoassay (EIA) reader for absorbance at a wavelength of 450 nm.

Antibodies against SARS-CoV-2, if present in the sample, will bind to the recombinant SARS-CoV-2 RBD protein and block the protein-protein interaction between HRP-conjugated RBD and the cell receptor protein hACE2. The absorbance of the sample is inversely proportional to the titer of neutralizing antibodies against SARS-CoV-2.

After the test validity as per the manufacturer's instruction, the inhibition rate for each sample was calculated. Results were reported positive if ≥30% inhibition was noted. About <30% inhibition was reported as negative. The sensitivity and specificity of the kit are 96.99% and 100%, respectively.

The SARS-CoV-2 Ab positivity rate was expressed in percentage and was correlated with gender and age groups. The Ab-positive participants were categorized based on the NAT in Table 2.

**Table 2:** Categorization based on the neutralizing antibody titer.

Category	Neutralizing antibody titer levels (percentage)
Category 1	Neutralizing antibody titer <50%
Category 2	Neutralizing antibody titer between 50% and 90%
Category 3	Neutralizing antibody titer >90%

Statistical analysis

Sex values were expressed in percentage. Age was expressed as the mean, and lab investigation was expressed as the mean±standard deviation. Antibody positivity and NAT were expressed in percentage. These were again correlated with age and sex and applied chi-square testing significance. Statistical Package for the Social Sciences (SPSS, IBM Corp., New York, USA) was used for statistical analysis, and p<0.05 was considered statistically significant.

## Results

A total of 58 participants (49 healthy participants and nine RA patients) were recruited from November 2021 to February 2022. Out of 58 participants, 40 were males, nine were females among healthy participants, and one male and eight females in the RA group were enrolled. The demographic details and baseline lab investigations were presented in Table 3.

**Table 3:** Demographic and baseline characteristics of participants. CRP: C-reactive protein, ESR: erythrocyte sedimentation rate, DAS-ESR 28 score: disease activity score-erythrocyte sedimentation rate, CBC: complete blood count, LFT: liver function test, SGOT: serum glutamic oxaloacetic transaminase, SGPT: serum glutamic pyruvic transaminase, ALP: alkaline phosphatase. All laboratory parameters are expressed in mean±SD.

Character		Control (n=49)	Test (n=9)
Male (n)		40	1
Female (n)		9	8
Mean age in years		34	49.8
CRP (mean)		Negative (<6 mg/L)	12.6
ESR		15.32±10.62 (n=31) (mm/h)	28.5±15
RA factor (mean)		Negative (<10 IU/mL)	53.3
DAS-ESR 28 score (mean)		-	4.2
CBC	Total WBC count	6467±2078 (thousands)	7261±1789
	RBC	5.01±0.79 (mill/cumm)	4.4±0.32
	Hb	14.31±2.7 (mg/dL)	12.24±2.5
LFT	Total bilirubin	0.77±0.49 (mg/dL)	0.38±0.14
	Total protein	7.5±0.78 gm/dL	7.03±0.7
	SGOT	24.8±6.76 (U/L)	18.1±3.85
	SGPT	20.95±3.85 (U/L)	15.2±7.32
	ALP	82.6±22.15 (U/L)	81.6±16.24
Serum creatinine		0.74±0.14 (mg/dL)	0.7±0.07
Blood urea		19.6±6.73 (mg/dL)	19±4.9

Among the RA patients, one participant was found to have COPD, and two participants with hypothyroidism. The mean age was 34 years (range: 20-65) for the control group and 49.8 years for RA patients. The neutralizing SARS-CoV-2 antibody test results showed positivity of 83.6%, with the predominant positive rate in males (56%) among the healthy participants, whereas most RA patients were females and all showed positivity (100%) in the RA group. Neutralizing antibody titer (NAT) >50% was observed in 65% and 88.8% of young adults aged between 20 and 39 years among the healthy group and RA group, respectively.

There was no significant difference in age and gender-specific positivity for SARS-CoV-2 neutralizing antibodies and neutralizing antibody titer among healthy participants (Table 4).

**Table 4:** Correlation of age and gender for SARS-CoV-2 neutralizing antibodies in pre-vaccinated healthy participants. Statistically significant: p<0.05. SARS-CoV-2: severe acute respiratory syndrome coronavirus 2.

Healthy participants							
Category	SARS-CoV-2 neutralizing antibodies (n=49)		P-value	Neutralizing antibody titer (n=41)			P-value
Age group	Positive (n=41)	Negative (n=8)		<50% (n=12)	50-90% (n=20)	>90% (n=9)	
Young adults (20-39 years)	35	7	0.87	10	17	6	0.49
Middle-aged (40-59 years)	6	1		2	3	3	
Gender-specific	Positive (n=41)	Negative (n=8)	P-value	<50% (n=12)	50-90% (n=20)	>90% (n=9)	P-value
Male	33	7	0.55	10	17	6	0.49
Female	8	1		2	3	3	
Total	41 (83.6%)	8 (18.4%)	P>0.05	12 (29.3%)	20 (48.8%)	9 (21.9%)	P>0.05

**Figure 1:**
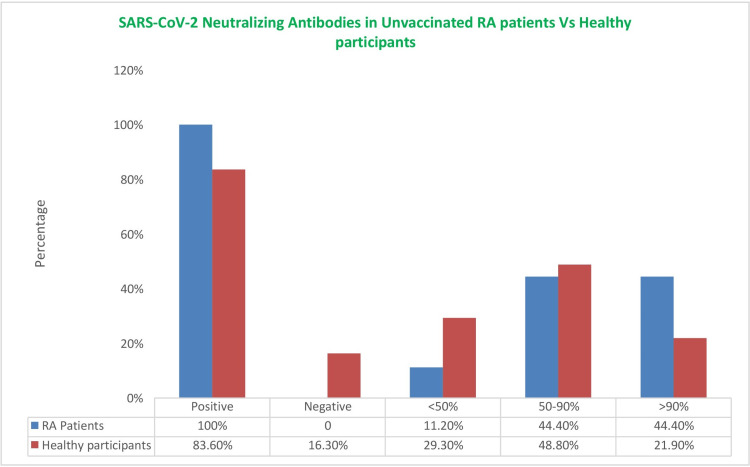
SARS-CoV-2 neutralizing antibodies in pre-vaccinated RA patients and healthy participants. Healthy participants: n=49, rheumatoid arthritis (RA) patients: n=9. SARS-CoV-2: severe acute respiratory syndrome coronavirus 2.

Comparison of Ab positivity rate and NAT between healthy participants and RA patients also showed no significant difference, and the p-value for SARS-CoV-2 neutralizing antibodies and the NAT was 0.53 and 0.30, respectively (Figure 1).

## Discussion

The present study was conducted on 58 participants to know their pre-vaccination anti-spike antibody status. The recruitment happened around November 2022 and January 2023. We were able to recruit an adequate control population as per the proposed protocol, but not the RA patients. Our hospital was recently established; hence, we were not able to recruit adequate RA patients. Control participants were healthy participants without any co-morbidities. RA patients had elevated ESR, CRP, and DAS-28 scores. It is not possible to evaluate the disease activity in RA patients using a single parameter. Hence, DAS-28, which takes multiple parameters such as swelling and tenderness of the joints, CRP or ESR, and the general condition of the patient into consideration, has been used most commonly for evaluating the disease activity in this patient group. The DAS-28 score was calculated online using the ESR values. In our study, the mean score of 4.2 indicates that the disease severity is moderate.

A humoral immune response against SARS-CoV-2 takes place in most patients during the initial weeks of infection [4]. The immune response against COVID-19 produces antibodies against spike proteins and nucleocapsids. These antibodies can be of neutralizing or non-neutralizing type [5]. Just like other viral infections, three types of antibodies, such as IgG, IgM, and IgA, can be used to detect the body’s immunological response to the SARS-CoV-2 infection. IgM antibodies start appearing within a short period of time after the onset of infection, while IgG starts to appear in a more delayed timescale. It has been reported that more than 90% of the patients develop specific immunoglobulin M (IgM) and IgG by day 10 and 100% by day 19. Seroconversion from IgM to IgG is crucial for the clearance of the COVID-19 infection [6-8]. The median seroconversion time for total antibody (Ab), IgM, and IgG is 11 days, 12 days, and 14 days, respectively[9]*.*

On average, 33% to 41% of asymptomatic individuals had shown positivity for anti-SARS-CoV-2 antibodies and a higher antibody positivity rate in asymptomatic individuals than in symptomatic patients [10,11]. The tests for SARS-CoV-2 antibodies may help healthcare providers to identify the presence of antibodies to the SARS-CoV-2 virus, indicating a prior infection with the virus. At present, the Center for Disease Control and Prevention (CDC) and the United States Food and Drug Administration (USFDA) are not recommending antibody testing for the assessment of immunity to SARS-CoV-2 following COVID-19 vaccination or the need for vaccination in an unvaccinated person [12,13].

In our study also the majority of the healthy volunteers who gave a history of no COVID-19 infection in the recent three months have shown positivity for anti-spike antibodies. In spite of the fact that the natural infection may provide a certain level of immunity, there is an inter-individual variability in the duration and strength of this immunity. Vaccination against SARS-CoV-2 has shown to develop higher consistent and long-lasting immunity compared to natural infection.

Additionally, the virus can come up with new variants due to its mutations in due course of time, which cannot be tackled by natural immunity. The COVID-19 vaccines are developed in such a way that they shall provide protection against these new variants and are updated from time to time. Hence, the COVID-19 vaccine is recommended irrespective of the anti-spike antibody status, either due to natural infection or due to herd immunity.

Herd immunity plays a crucial role in long-term protection against the infection, which can be achieved either by direct exposure to the infection or by covering the major population with vaccination. The World Health Organization (WHO) suggests the COVID-19 mass vaccination campaigns achieve herd immunity [14,15].

Our study reported that overall, 83.6% were positive for SARS-CoV-2 NAbs. These NAbs are thought to be essential for recovery and defense against viral diseases, but the duration of protection by the SARS-CoV-2 NAb against re-infection is poorly understood [16-19].

It has been reported that 67 out of 94 (71.3%) samples were positive for anti-spike receptor binding domain (RBD) antibodies when measured after the second wave in India, indicating prior exposure to COVID-19 [20].

The antibody titer range was 50-90% in the majority of the population (48%), which is relatively higher and can be attributable to substantial COVID-19 vaccine coverage leading to the development of herd immunity [21] and comparable with Oran et al. and Kumar et al. study reports [10,11]. Our study showed no statistically significant gender difference for antibody positivity rate and for antibody titer, which was similar to the findings by Charlton et al. [22], but contrary findings were observed with respect to age, where the younger population has the highest seropositivity than the older population.

Recent evidence shows that, in COVID-19 infection, there is an increase in the plasma cytokine levels, especially IL-6, TNF alpha, and chemokines, which have a positive correlation with COVID-19 severity. In RA patients, inflammation is promoted with an increase of these cytokines and chemokines. Hence, it is evident that the pathophysiological changes of both COVID-19 and RA have an identical mechanism leading to deranged immune reactions, causing exaggerated action of the cytokine-chemokine axis. Several studies have shown the presence of autoantibodies in patients suffering from COVID-19 infection, especially anti-cardiolipin, anti-β2-glycoprotein I, and antinuclear antibodies. Anti-citrullinated protein antibodies (ACPAs) and exacerbation of features of RA during the infection have also been noted, but the exact mechanism is still unclear.

It is important to understand how the immune system of RA patients responds to the vaccination, especially considering the nature of the disease [20]. Despite the immune-compromised nature of the disease, RA patients are advised to take the COVID-19 vaccine unless contraindicated otherwise while continuing their immunosuppressant drugs [23-25]. The nine RA patients showed positivity for anti-spike antibodies, which may be due to an immunological alteration caused by RA or due to the treatment of RA. There was no difference observed in the positivity for SARS-CoV-2 antibodies between RA and healthy participants.

Limitations

The study presents the pre-vaccination anti-spike antibody status only and not the follow-up antibody status after vaccination. As our institute is newly established, we were able to recruit only nine RA patients for this study.

## Conclusions

Our study showed 84% positivity for anti-spike SARS-CoV-2 antibodies around the third wave. The majority of young male participants had high neutralizing antibody titers, and the highest percentage was seen in RA patients. The probable reason for the SARS-CoV-2 antibody positivity before vaccination was either asymptomatic infection or herd immunity.
